# Supplementary material for: Identification of photoperiod-induced specific miRNAs in the adrenal glands of Sunite sheep (Ovis aries)
Source: Front Vet Sci. 2022 Jul 22;9:888207. doi: 10.3389/fvets.2022.888207 (PMC9354845; doi:10.3389/fvets.2022.888207)
Supplement: Supplementary file 1 [file Data_Sheet_1.ZIP › Supplementary file/miRDeep2 file.zip!/miRDeep2 file/Know_Structure/SPLP42A2/oar-mir-143.pdf]

miRBase precursor : oar-mir-143
Total read count : 535741
oar-miR-143 read count : 532324
remaining reads : 3417

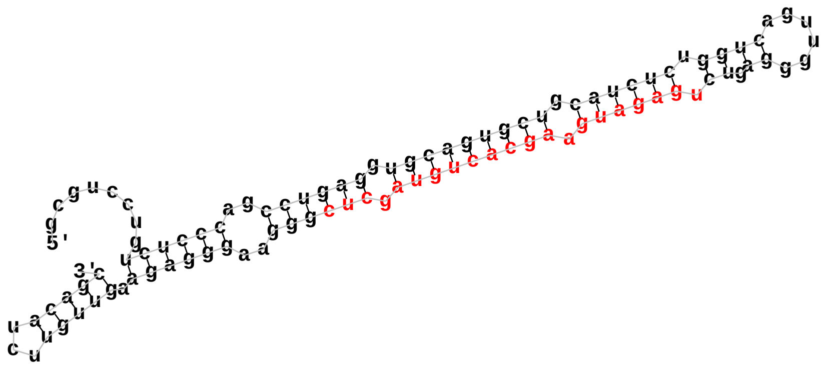

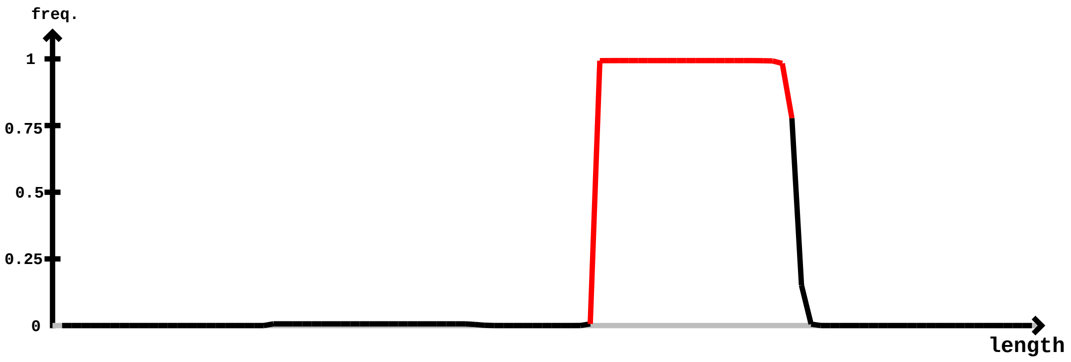

oar-miR-143

5'- gcguccugucucccagccugaggugcagugcugcaucucuggucaguugggagucugagaugaagcacuguagcucgggaagggagaaguuguucuacagc -3'	exp		
........((((((..((((((.(((((((((.((((((.((((......)).)).)))))).))))))))).))))))..)))))).(((((...)))))	reads	mm	sample
....................aggugcagugcugcaucucuggu..	4	0	seq
.....................ggugcagugcugcaucucu...	3	0	seq
.....................ggugcagugcugcaucucug..	25	0	seq
.....................ggugAagugcugcaucucug..	1	1	seq
.....................ggugcagugcugcaucucugg...	1184	0	seq
.....................ggugcagugcugcaucucuggu..	1599	0	seq
.....................ggugcagugcugcaucucugguc...	550	0	seq
.....................ggugcagugcugcaucucugguca..	44	0	seq
.....................ggugcagugcugcaucucuggucag...	1	0	seq
.....................ggugcagugcugcaucucuggucaguu...	3	0	seq
......................gugcagugcugcaucucuggu..	1	0	seq
....ucaguugggagucugagaugaagcacuguagcuc.........................	1	0	seq
....gucugagaugaagcacuguagcuc.........................	1	0	seq
....ucugagaugaagcacuguag............................	1	0	seq
....ucugagaugaagcacuguagc...........................	4	0	seq
....ucugagaugaagcacuguagcu..........................	9	0	seq
....ucugagaugaagcacuguagcuc.........................	71	0	seq
....ucugagaugaagcacuguagcucg........................	2	0	seq
....cugagaugaagcacugu..............................	4	0	seq
....cugagaugaagcacugG..............................	1	1	seq
....cugagaugaagcacuguG.............................	1	1	seq
....cugagaugaagcacugua.............................	2	0	seq
....cugagaugaagcacuguag............................	4	0	seq
....cugagaugaagcacuguagc...........................	59	0	seq
....cugagaugaagcacuguagcu..........................	776	0	seq
....cugagaugaagcacuguagcuc.........................	2229	0	seq
....cugagaugaagcacuguagcucg........................	209	0	seq
....cugagaugaagcacuguagcucgg.......................	2	0	seq
....ugagaugaagUacugua.............................	7	1	seq
....ugagaugaagcGcugua.............................	1	1	seq
....ugagaugaagcacuUua.............................	1	1	seq
....ugagaugaagcacuguU.............................	5	1	seq
....ugagaugaagcacugua.............................	255	0	seq
....ugagaugaagcacuCua.............................	1	1	seq
....ugagGugaagcacugua.............................	2	1	seq

oar-miR-143

gcguccugucucccagccugaggugcagugcugcaucucuggucaguugggagucugagaugaagcacuguagcucgggaagggagaaguuguucuacagc			
....ugagaugaagcaUugua.............................	1	1	seq
....ugagaugGagcacugua.............................	1	1	seq
....ugagaugaagcacuguG.............................	14	1	seq
....ugaCaugaagcacugua.............................	7	1	seq
....ugagauAaagcacuguag............................	1	1	seq
....ugagauUaagcacuguag............................	1	1	seq
....ugagaugaagcacuguag............................	383	0	seq
....ugagaugaagcacuguagc...........................	5014	0	seq
....ugagaugaagcacuguagcu..........................	109163	0	seq
....ugagaugaagcacuguagcuc.........................	333216	0	seq
....ugagaugaagcacuguagcucg........................	77804	0	seq
....ugagaugaagcacuguagcucgg.......................	2830	0	seq
....ugagaugaagcacuguagcucggg......................	33	0	seq
....ugagaugaagcacuguagcucggga.....................	1	0	seq
....gagaugaagcacuguagc...........................	1	0	seq
....gagaugaagcacuguagcu..........................	18	0	seq
....gagaugaagcacuguagcuc.........................	102	0	seq
....gagaugaagcacuguagcucg........................	27	0	seq
....agaugaagcacuguagcu..........................	2	0	seq
....agaugaagcacuguagcuc.........................	2	0	seq
....agaugaagcacuguagcucg........................	2	0	seq
....agaugaagcacuguagcucgg.......................	19	0	seq
....agaugaagcacuguagcucggg......................	10	0	seq
....gaugaagcacuguagcu..........................	1	0	seq
....gaugaagcacuguagcuc.........................	1	0	seq
....gaugaagcacuguagcucg........................	1	0	seq
....gaugaagcacuguagcucgg.......................	12	0	seq
....gaugaagcacuguagcucggg......................	8	0	seq
....gaugaagcacuguagcucggga.....................	3	0	seq
